# Antimicrobial resistance in *Neisseria gonorrhoeae*: Global surveillance and a call for international collaborative action

**DOI:** 10.1371/journal.pmed.1002344

**Published:** 2017-07-07

**Authors:** Teodora Wi, Monica M. Lahra, Francis Ndowa, Manju Bala, Jo-Anne R. Dillon, Pilar Ramon-Pardo, Sergey R. Eremin, Gail Bolan, Magnus Unemo

**Affiliations:** 1 Department of Reproductive Health and Research, World Health Organization, Geneva, Switzerland; 2 World Health Organization Collaborating Centre for Sexually Transmitted Diseases, New South Wales Health Pathology, Sydney, Australia; 3 School of Medical Sciences, The University of New South Wales, Sydney, Australia; 4 Skin and Genitourinary Medicine Clinic, Harare, Zimbabwe; 5 Apex Regional STD Teaching, Training & Research Centre, VMMC and Safdarjung Hospital, New Delhi, India; 6 University of Saskatchewan, Saskatoon, Saskatchewan, Canada; 7 Communicable Disease Analysis, World Health Organization, Washington, D.C., United States of America; 8 Antimicrobial Resistance Secretariat, World Health Organization, Geneva, Switzerland; 9 Division of STD Prevention, Centers for Disease Control and Prevention, Georgia, Atlanta, United States of America; 10 World Health Organization Collaborating Centre for Gonorrhoea and other STIs, Örebro University, Örebro, Sweden

## Abstract

In a Policy Forum, Teodora Wi and colleagues discuss the challenges of antimicrobial resistance in gonococci.

**Summary points**

- Antimicrobial resistance (AMR) in *Neisseria gonorrhoeae* seriously compromises the management and control of gonorrhea.
- In vitro and clinical resistance in *N*. *gonorrhoeae* to the extended-spectrum cephalosporins (ESCs), the last-line treatment for gonorrhea, have been reported from many, particularly well-resourced, settings globally.
- The World Health Organization (WHO) Global Gonococcal Antimicrobial Surveillance Programme (GASP) is key to monitoring AMR trends, identifying emerging AMR, and informing regular refinements of treatment guidelines and public health policy globally.
- Enhanced international collaborative actions are crucial for the control of gonorrhea, including improved prevention, early diagnosis (development of accurate, rapid, point-of-care tests), partner management, and enhanced surveillance (including population-based surveillance and surveillance of treatment failures and antimicrobial use).
- Rapid, accurate, point-of-care diagnostic tests (which would ideally predict AMR and/or antimicrobial susceptibility), new therapeutic drugs, and a gonococcal vaccine—which will ultimately be the only sustainable way to achieve gonorrhea control—are needed.

## Introduction

Gonorrhea is a sexually transmitted infection (STI) caused by *Neisseria gonorrhoeae* (gonococcus), and it is a major public health priority globally. In 2012, the World Health Organization (WHO) estimated that there were 78 million cases among adults worldwide, including 35.2 million in the WHO Western Pacific Region, 11.4 million in the Southeast Asian Region, 11.4 million in the African Region, 11 million in the Region of the Americas, 4.7 million in the European Region, and 4.5 million in the Eastern Mediterranean Region [1]. According to the 2013 Global Burden of Disease Study, gonorrhea is responsible for 225,400 years lived with disability (YLD) per year and 313,900 disability-adjusted life years (DALYs) [2,3]. The complications of gonorrhea disproportionally affect women and include pelvic inflammatory disease, ectopic pregnancy, and infertility, as well as increased transmission and acquisition of HIV [4–6].

Widespread antimicrobial resistance (AMR) in highly variable strains of *N*. *gonorrhoeae* has continuously compromised the management and control of gonorrhea. Because of widespread AMR, the persistence of AMR determinants in gonococci, and the unavailability of diagnostic tests that provide AMR results at the time of treatment, clinicians resort to empiric treatment for gonorrhea. Since the introduction of antimicrobial treatment, resistance has rapidly emerged to sulphonamides, penicillins, tetracyclines, macrolides, fluoroquinolones, and early-generation cephalosporins. Currently, in most countries, the injectable extended-spectrum cephalosporin (ESC) ceftriaxone is the only remaining empiric monotherapy for gonorrhea. However, gonococcal in vitro resistance and/or treatment failures to the last-line oral ESC cefixime—and, more rarely, to ceftriaxone—have been verified in many countries [5–8]. Consequently, dual antimicrobial therapy, mainly ceftriaxone plus azithromycin, is recommended [9–13].

In 2016, the United Nations (UN) World Health Assembly endorsed the WHO global health sector strategy on sexually transmitted infections 2016–2021. One of the major targets is a 90% reduction in the incidence of gonorrhea globally [14]; to achieve this goal, gonococcal AMR needs to be addressed. In 2012, WHO launched a global action plan to control the spread and impact of gonococcal AMR [5,15]; key priorities are summarized in Box 1. This plan is linked to the WHO global action plan on AMR, adopted by the World Health Assembly in 2015 [16], which was reaffirmed during the UN General Assembly High-level Meeting on AMR in September 2016.

**Box 1. Key priorities of the World Health Organization (WHO) global action plan to control the spread and impact of antimicrobial resistance (AMR) in *Neisseria gonorrhoeae* [5]**

- Advocacy for increased awareness on the correct use of antibiotics among healthcare providers and the consumer, particularly in key populations, including men who have sex with men (MSM) and sex workers.
- Effective prevention, diagnosis, and control of gonococcal infections, using prevention messages and prevention interventions, and recommended adequate diagnosis and appropriate treatment regimens.
- Systematic monitoring of treatment failures by developing a standard case definition of treatment failure and protocols for verification, reporting, and management of treatment failure.
- Effective drug regulations and prescription policies.
- Strengthened AMR surveillance, especially in countries with a high burden of gonococcal infections, other sexually transmitted infections (STIs), and HIV.
- Capacity building to establish regional networks of laboratories to perform gonococcal culture with good-quality control mechanisms.
- Research into new molecular methods for monitoring and detecting AMR and development of new treatment options.
- Research into, and identification of, alternate effective treatment regimen(s) and vaccine(s) for gonococcal infections.

## WHO GASP

The WHO Global Gonococcal Antimicrobial Surveillance Programme (WHO GASP), a collaborative global network of regional and subregional reference laboratories, was initiated in 1990 to monitor gonococcal AMR worldwide [17]. WHO GASP data have since then informed revisions of global, regional, and national gonorrhea treatment guidelines, as well as public health strategies and policies developed by WHO and other organizations. WHO recommends that treatment guidelines are refined based on data from recent and quality-assured gonococcal AMR surveillance and that the use of an antimicrobial in empiric treatment is discontinued when the rates of therapeutic failures and/or AMR reach a level of 5% [5,18]. Since 2009, WHO has substantially strengthened the WHO GASP, which is coordinated by regional coordinating centers (“focal points”) (see S1 Table). To ensure quality-assured, valid, and comparable data among countries, regional focal points provide technical support and training in countries to strengthen laboratory capacities (e.g., for sample collection and transport, gonococcal culturing, preservations of strains, AMR testing), to conduct a GASP external quality assurance (EQA) program, and to curate, update, and distribute the WHO gonococcal reference strains for EQA and internal quality control (QC) [19,20]. The 2016 WHO reference strains can also be used for QC in phenotypic and molecular diagnostics, molecular AMR prediction, molecular epidemiology, and as fully characterized reference genomes in whole-genome sequencing analysis [20]. The WHO GASP works in close collaboration with other international and national quality-assured GASPs, including Euro-GASP [21,22], United States Gonococcal Isolate Surveillance Project (GISP; https://www.cdc.gov/std/gisp/) [23,24], Canadian GASP [25], Australian Gonococcal Surveillance Programme (AGSP) [26], and United Kingdom Gonococcal Resistance to Antimicrobials Surveillance Programme (UK GRASP) [27].

### AMR data reporting by countries participating in WHO GASP

There are significant variations between WHO regions with regard to the proportion of countries participating in the WHO GASP, which antimicrobials are monitored, the AMR testing methods used, and the level of quality assurance (QA). The methodologies used, including QA methods, are described in S1 Text [19–34].

The cumulative number of countries reporting gonococcal AMR data for any antimicrobial increased from 56 in 2009 to 77 in 2014. However, the number of countries reporting gonococcal AMR data for at least 1 antimicrobial each year declined, from 56 countries in 2009 to 52 countries in 2014. The WHO European Region [28] and Western Pacific Region GASPs [29] include many countries that consistently report on AMR, and many Latin American countries (LACs) also have a long tradition of consistently reporting gonococcal AMR [30,31]. Countries in the WHO African Region [32] and Eastern Mediterranean Region are the least represented in terms of gonococcal AMR reporting. The number of countries reporting data on *N*. *gonorrhoeae* antimicrobial susceptibility to ESCs, azithromycin, and ciprofloxacin in 2009–2014 are detailed in S2 Table.

### Recent findings from the WHO GASP

WHO GASP data from 2009–2014 showed continued widespread resistance to penicillin, tetracycline, and ciprofloxacin; increasing resistance to azithromycin; and emergence of decreased susceptibility and resistance to ESCs. Of countries monitoring susceptibility to ciprofloxacin (*n* = 72), azithromycin (*n* = 58), and ESCs (*n* = 77), 97%, 81%, and 66% of countries, respectively, described resistant isolates (decreased susceptibility and resistance to ESCs were combined due to the different breakpoints used) for at least 1 year from 2009–2014 (Table 1).

**Table 1:** The number of countries in different WHO regions reporting gonococcal isolates with resistance to azithromycin and ciprofloxacin, and decreased susceptibility or resistance to ESCs (cefixime and/or ceftriaxone) for at least 1 year from 2009 to 2014.

Resistance of gonococcal isolates to antimicrobials	WHO regions							Countries (%) reporting resistance/ decreased susceptibility
	Africa	Americas	Eastern Mediterranean	Europe	Southeast Asia	Western Pacific	Total	
**ESCs**								
Countries reporting	9	16	3	27	6	16	77	
≥5% resistance^a^	1	0	0	15	4	6	26	51 (66%)
<5% resistance	2	6	0	8	1	8	25	
Full susceptibility	6	10	3	4	1	2	26	
**Azithromycin**								
Countries reporting	3	7	1	26	6	15	58	
≥5% resistance^a^	3	2	0	21	1	2	29	47 (81%)
<5% resistance	0	4	0	3	4	7	18	
Full susceptibility	0	1	1	2	1	6	11	
**Ciprofloxacin**								
Countries reporting	8	16	1	26	6	15	72	
>90% resistance^b^	0	1	1	3	4	5	14	70 (97%)
≥5% resistance^a^	6	14	0	23	2	7	52	
<5% resistance	0	1	0	0	0	3	4	
Full susceptibility	2	0	0	0	0	0	2	

^a^ Resistance level at which WHO recommends that the use of an antimicrobial in empiric treatment is discontinued.

^b^ An arbitrary resistance level was included to show that the resistance levels to ciprofloxacin are extremely high in many parts of the world, particularly in the WHO Southeast Asian Region and Western Pacific Region.

ESC, Extended-spectrum cephalosporins; WHO, World Health Organization

**Fig 1:**
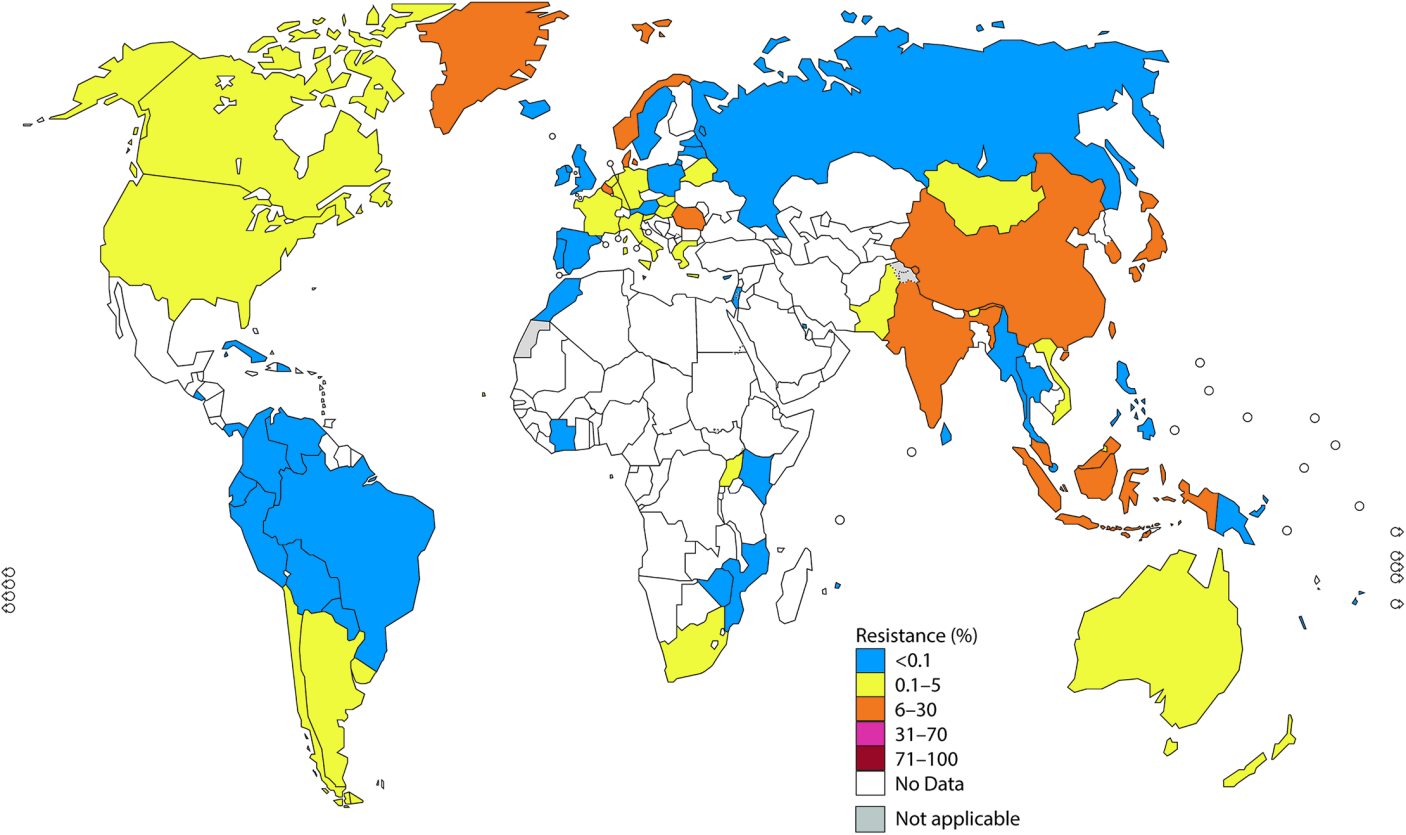
The percentage (%) of isolates with decreased susceptibility or resistance to extended-spectrum cephalosporin (ESC) (cefixime and/or ceftriaxone) according to the most recent World Health Organization (WHO) Gonococcal Antimicrobial Surveillance Programme (GASP) data (2014 for most countries, but for a few countries, only 2011–2013 data were available). Note: The areas in grey are disputed territories (e.g., Western Sahara, Jammu, and Kashmir), and no antimicrobial resistance (AMR) data are available from these regions.

**Fig 2:**
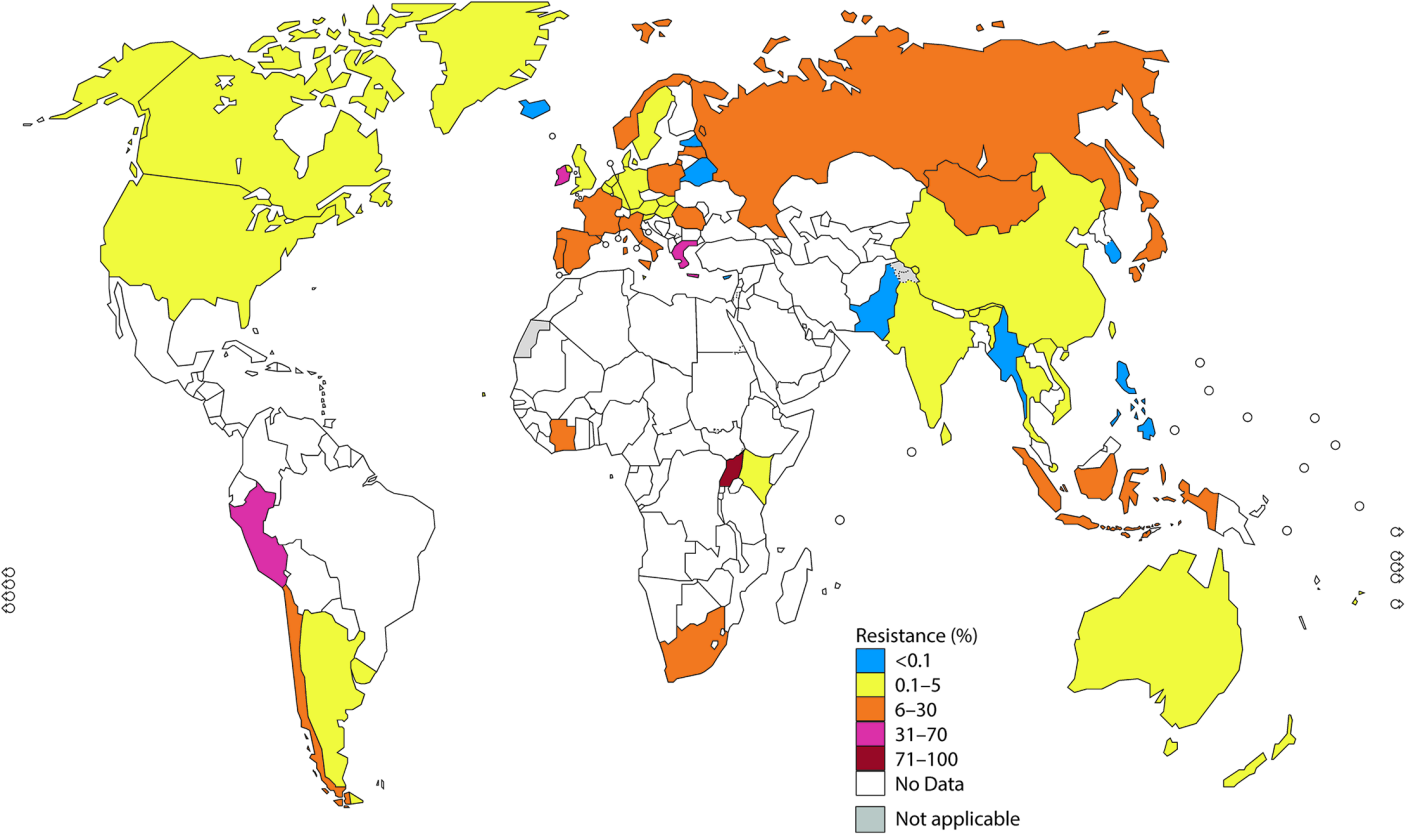
The percentage (%) of isolates with resistance to azithromycin according to the most recent World Health Organization (WHO) Gonococcal Antimicrobial Surveillance Programme (GASP) data (2014 for most countries, but for a few countries, only 2011–2013 data were available). Note: The areas in grey are disputed territories (e.g., Western Sahara, Jammu, and Kashmir), and no antimicrobial resistance (AMR) data are available from these regions.

**Fig 3:**
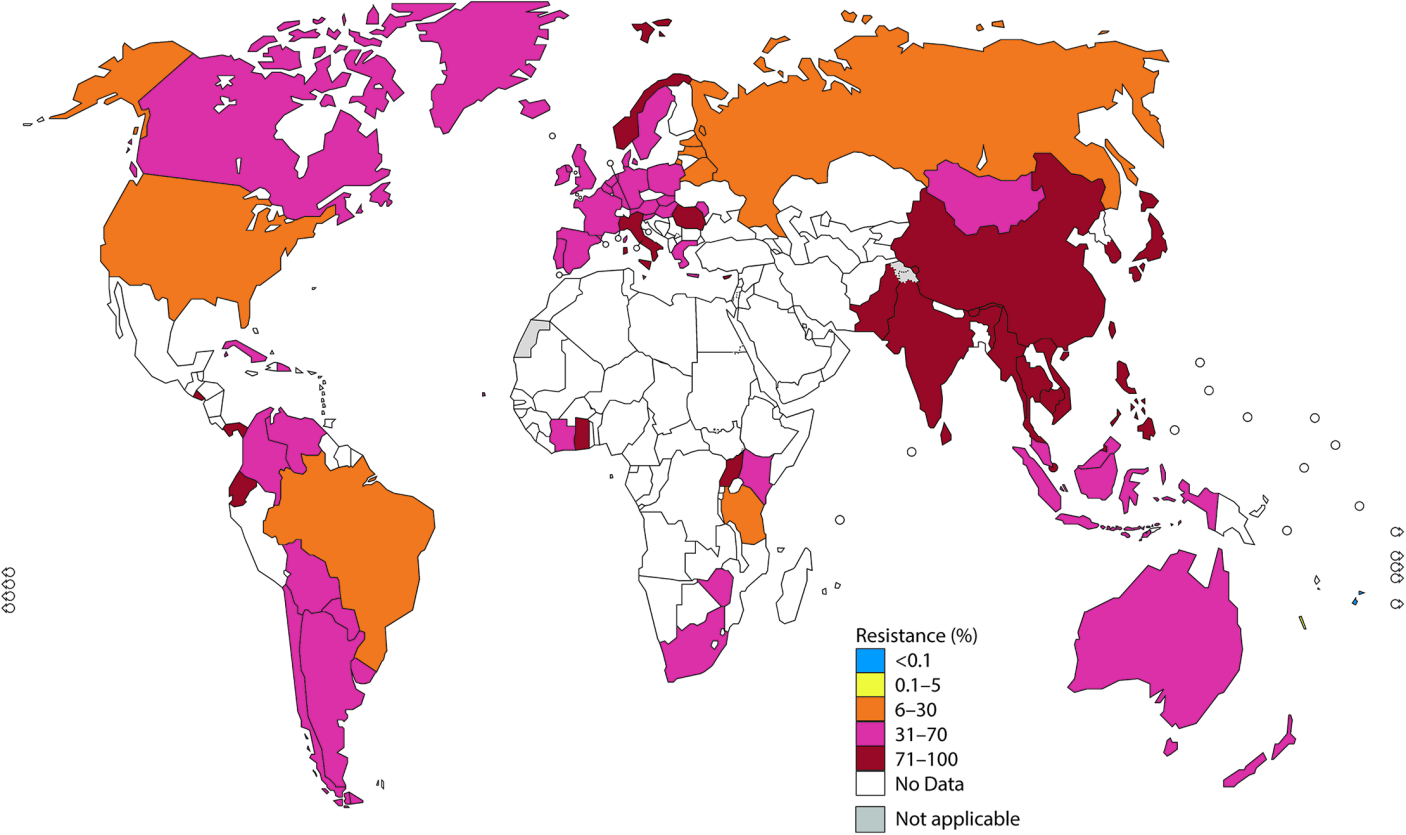
The percentage (%) of isolates with resistance to ciprofloxacin according to the most recent World Health Organization (WHO) Gonococcal Antimicrobial Surveillance Programme (GASP) data (2014 for most countries, but for a few countries, only 2011–2013 data were available). Note: The areas in grey are disputed territories (e.g., Western Sahara, Jammu, and Kashmir), and no antimicrobial resistance (AMR) data are available from these regions.

In Figs 1–3, the most recent WHO GASP data from each country are summarized.

#### ESCs

In 2014, 45 countries reported data on gonococcal susceptibility to ESCs, and 51% (23/45) reported isolates with decreased susceptibility or resistance to ESCs. Notably, resistance level to cefixime (oral) was substantially higher compared to ceftriaxone (intramuscular) because cefixime is less effective [6,7,22,35].

Twenty-three countries in the WHO European Region reported on gonococcal AMR. In the Euro-GASP [21,22], cefixime is tested and accounted for nearly all the ESC resistance in the WHO European Region in 2014 (Fig 1). Two percent of isolates in the Euro-GASP, detected in 43% (10/23) of the reporting countries, showed resistance to cefixime in 2014. Seventeen percent of countries (4 countries: Belgium, Denmark, Greece, and Norway) reported ≥5% resistance, and 26% of countries (6/23) reported <5% cefixime resistance. No resistance to cefixime was reported in 57% (13/23) of countries; however, 10 of these have reported cefixime resistance in previous years. Resistance to ceftriaxone was only identified in 0.2% (5/2,151) of isolates (from Germany, Greece, and Norway) [35].

In the WHO Western Pacific Region, decreased susceptibility or resistance to ceftriaxone was reported by 71% (5/7) of settings reporting ceftriaxone susceptibility data in 2014, 3 of which (Hong Kong, Japan, and Korea) reported decreased susceptibility or resistance in ≥5% of isolates. Prior to this, China, Mongolia, and Tonga have reported ≥5% of isolates with decreased susceptibility or resistance to ceftriaxone (in Tonga, 1 of only 4 tested isolates showed decreased susceptibility with the disc diffusion method and was not, as recommended (S1 Text), verified by minimum inhibitory concentration [MIC] determination). Only 2 countries (New Caledonia and the Philippines) reported that all isolates were susceptible to ceftriaxone from 2009 to 2014.

In the WHO Southeast Asian Region, decreased susceptibility or resistance to ceftriaxone was noted by 50% (3/6) of the countries (Bhutan, India, and Indonesia) reporting ceftriaxone susceptibility data in 2014; 33% (2/6; India and Indonesia) reported ≥5% decreased susceptibility or resistance. From 2009 to 2014, 83% (5/6) of countries reported isolates with decreased susceptibility or resistance to ceftriaxone, and 50% (3/6) reported ≥5% decreased susceptibility or resistance.

In the WHO Region of the Americas, decreased susceptibility or resistance to ceftriaxone was described in 40% of 10 reporting countries in 2014 (Argentina, Bolivia [disc diffusion results not verified by MIC determination], Canada, and the US [24]). The levels of decreased susceptibility or resistance to ceftriaxone in Canada and the US [24] were 1.1% and 0.4%, respectively. Decreased susceptibility or resistance to cefixime was noted in Argentina, Chile, and Uruguay in 2013–2014.

Surveillance for susceptibility to ceftriaxone, as well as other antimicrobials, is very limited in the WHO African Region and the WHO Eastern Mediterranean Region. In 2014, only 9% (4/47) of countries in the WHO African Region and only Pakistan in the Eastern Mediterranean Region reported on ceftriaxone susceptibility (4.9% decreased susceptibility or resistance). Of note, only 15% (7/47) of WHO African Region countries and 14% (3/22) of WHO Eastern Mediterranean Region countries have reported on ceftriaxone susceptibility for at least 1 year from 2009–2014. Only 2 of these countries (South Africa and Uganda) have reported any decreased susceptibility or resistance to ceftriaxone.

#### Azithromycin (macrolides)

In 2014, gonococcal susceptibility to azithromycin was surveyed in 45 WHO GASP countries. Twenty-three of 31 countries (74%; 14 countries with = 5% resistance) and 35 of 45 countries (78%; 13 countries with ≥5% resistance) reported azithromycin-resistant isolates in 2009 and 2014, respectively.

Among 23 reporting WHO European Region countries, 87% (20/23) reported azithromycin-resistant isolates, including 39% (9/23) reporting ≥5% resistance and 22% (5/23) reporting >10% resistance. Only 13% (3/23) of countries reported no azithromycin-resistant isolates in 2014; however, 1 of these countries previously reported azithromycin resistance [35].

In the WHO Western Pacific Region, 78% (7/9) of countries reported azithromycin-resistant isolates, with 3 settings reporting ≥5% resistance (Japan, Hong Kong, and Mongolia), 4 countries reporting <5% resistance (Australia, New Zealand, Singapore, and Vietnam), and 2 countries reporting no resistance (Korea and the Philippines).

In the WHO Southeast Asian Region, of the 6 countries reporting in 2014, 1 country (Indonesia) reported ≥5% resistance to azithromycin, 4 (67%) reported <5% resistance, and 1 country (Myanmar) reported no resistance (but only 4 isolates were tested).

In the WHO Region of the Americas, only 2 countries (US [24] and Canada) reported on azithromycin susceptibility in 2014, and 2.5% and 3.3% of isolates, respectively, were resistant to azithromycin.

In the WHO African Region, in 2014, only 3 out of 47 countries (6%; Côte d’Ivoire, Kenya, and Uganda) reported on azithromycin susceptibility, and all three had ≥5% resistance. Furthermore, South Africa reported ≥5% resistance in 2013. In the WHO Eastern Mediterranean Region, only Pakistan reported data regarding azithromycin susceptibility in 2014 (0% resistance).

#### Ciprofloxacin (fluoroquinolones)

High levels of ciprofloxacin resistance have been reported in a majority of countries globally, especially in the WHO Southeast Asia Region and the Western Pacific Region. In these WHO regions, nearly all countries have reported high rates of resistance, including 9 countries reporting >90% resistance. Notably, a few WHO Western Pacific Region countries (Fiji and New Caledonia) have reported less than 2% resistance to ciprofloxacin. The majority of countries in the WHO European Region, the Region of the Americas, and the African Region have reported high resistance rates, which are still significantly lower than those in Asia (Fig 3).

### Treatment failure with ESCs

Treatment failures with cefixime have been identified in Japan since the early 2000s [36,37], followed by verified treatment failures in Austria, Canada, France, Norway, South Africa, and the UK [38–44]. Rare treatment failures with ceftriaxone (250–1,000 mg single dose) administered for pharyngeal gonorrhea have been verified in Australia, Japan, Slovenia, and Sweden [45–51]. The first verified treatment failure with the UK-recommended dual therapy (ceftriaxone 500 mg plus azithromycin 1 g) was recently identified [52]. To date, 3 extensively drug-resistant gonococcal strains with high-level resistance to ceftriaxone (“superbugs”) have also been reported—in France, Japan, and Spain [41,45,53].

The vast majority of verified treatment failures are from well-resourced countries. Accordingly, these reports do not reflect the true global public health burden of ESC treatment failures, since surveillance data from resource-constrained settings are scarce. As emphasized by WHO [5,33,54], it is essential to strengthen the surveillance—including verification and follow-up—of treatment failures.

## Discussion

### Challenges and opportunities with GASPs

Gonococcal AMR is a major problem globally, but the AMR situation varies in different parts of the world and changes over time. Sustained and quality-assured GASPs are essential but are very challenging to operate. Worryingly, gonococcal AMR surveillance remains lacking or exceedingly limited in many settings worldwide, e.g., Eastern Europe, Central Asia, parts of Latin America (including the Caribbean), the WHO Eastern Mediterranean Region, and the WHO African Region. Many of these settings also have high rates of gonorrhea, lack of (or suboptimal) diagnosis, over-the-counter access to antimicrobials without prescription, and limited access to optimal antimicrobial treatment, such as high-quality ceftriaxone or, ideally, dual therapy with ESC plus azithromycin. These factors create the perfect conditions for rapid emergence and spread of gonococcal AMR [5,6,28]. Accordingly, it is imperative to substantially strengthen and expand GASPs worldwide.

There are many microbiological, epidemiologic, and programmatic difficulties to the achievement of high-quality, standardized, and comparable AMR data. Between different countries and WHO regions, the number of isolates examined varies significantly. Almost half of the WHO GASP countries do not have sufficient sample sizes (approximately 100 gonococcal isolates per year) to detect a 5% AMR level with high statistical confidence [5,18,33], nor have they collected samples from representative populations. This is a result of syndromic management of STIs used in resource-constrained settings and limited laboratory capacity and capability. Meanwhile, in well-resourced settings, nucleic acid amplification tests (NAATs) have replaced gonococcal culture for diagnosis. Both circumstances have resulted in limited specimen collection for culture and loss of capability to perform culture and AMR testing of gonococci.

Situation analysis has revealed a lack of awareness of gonococcal AMR among policy-makers, clinicians, laboratory professionals, and patients in many settings [55]. Engagement of collaborators in sustainable GASPs is crucial. However, in addition to low awareness, other critical issues need to be addressed, such as data ownership, limited numbers of gonococcal-competent laboratories, clinical and laboratory training (in specimen collection, transport and preservation, and laboratory techniques such as culture and AMR testing), insufficient availability of appropriate laboratory tests and reagents (especially antimicrobials for testing), lack of or inadequate use of crucial QA components such as EQA and QC, loss of isolates during transport and storage (limited access to −70°C freezers, lyophilizators, lack of generators in case of power outages), and, most importantly, lack of sufficient funding.

Additionally, it is important to further strengthen well-established GASPs [21–27] by consistently testing crucial antimicrobials (e.g., ceftriaxone and azithromycin), increasing the number of isolates tested, improving the representativeness of isolates (e.g., geographically diverse isolates from both sexes, all risk groups, and all anatomical sites), improving the completeness of reporting of epidemiological variables, collecting additional epidemiological data, and gathering information on treatment and treatment outcomes where possible. In some sentinel countries (e.g., the Philippines and Thailand), WHO has initiated an enhanced GASP (EGASP) aiming to collect standardized and quality-assured epidemiological and clinical information linked to microbiological and AMR data, similar to Euro-GASP and US GISP [21–24]. These data should be collected in a timely manner in order to provide an early detection and warning system for the emergence of AMR. Furthermore, in the WHO GASP, the use of MIC determination methods is being expanded and it is recommended that all instances of ESC resistance identified by the disc diffusion method be confirmed by MIC determination.

In many settings, there is a lack of awareness among staff of national ministries of health and among healthcare professionals that continuous AMR surveillance and/or surveys should be the foundation of a national AMR action plan, should be part of routine diagnostics and/or surveillance, and should inform refinements of the recommended treatment algorithms. In settings in which clinical practices include syndromic management of STIs, this is especially important. Accordingly, AMR surveillance is not simply a research project. Thus, in most countries, it should not require approval from an ethical committee because the collection and antimicrobial-susceptibility testing of gonococcal isolates are part of standard care, and no patient identification information is made available in AMR surveillance. Finally, there are significant delays in the release of AMR data from the current GASPs, which limits their value as part of an early warning system for AMR emergence and limits their usefulness for informing prompt refinements of gonorrhea management guidelines and public health policy. Timely reporting by GASPs will require significant improvements in the procedures for release of country-specific AMR data (e.g., release directly to public health organizations before peer-review or through very timely peer-reviewed publications).

### Prevention and control of gonorrhea

Gonococcal AMR will only be effectively mitigated when the global gonorrhea burden is reduced. Improved prevention, management, and control of gonorrhea are imperative. Linking this to HIV and STI prevention in general will be essential, including education regarding symptomatic and asymptomatic STIs, promotion of safer sex behaviors including increased condom use, behavior change communication programs, enhanced partner notification and treatment, and expansion of targeted interventions for vulnerable populations (sex workers, men who have sex with men [MSM], adolescents, and STI patients and their sexual partners).

In the absence of a gonococcal vaccine, optimal public health control of gonorrhea will continue to rely on effective, accessible, affordable, and timely antimicrobial treatment in combination with prevention strategies, diagnostics (index cases and traced sexual contacts), and surveillance. Appropriate gonorrhea case management is essential to reduce unnecessary or incorrect antimicrobial treatment and development of AMR. In 2016, WHO published new guidelines for the treatment of gonorrhea [9], based on consultative review of all evidence available from clinical efficacy trials, pharmacokinetic and pharmacodynamic simulations, and in vitro AMR surveillance. WHO recommends that national public health programs adapt their national gonorrhea management guidelines based on local AMR prevalence. Where recent, local, and quality-assured gonococcal AMR data are lacking, WHO recommends dual antimicrobial therapy over monotherapy for people with symptomatic and asymptomatic urogenital, anorectal, or oropharyngeal gonorrhea (e.g., ceftriaxone 250 mg plus azithromycin 1 g, or cefixime 400 mg plus azithromycin 1 g) [9]. Increased detection and effective treatment of asymptomatic gonorrhea in general and pharyngeal gonorrhea in particular are critical, because these infections are potential gonococcal reservoirs in which AMR (especially ESC AMR) can emerge [6,56]. Oropharyngeal infections are prevalent, mostly asymptomatic, and more difficult to treat; accordingly, screening and treatment in high-risk patients are important.

### Research and development

During recent years, significant progress has been made in the understanding of mechanisms of gonococcal pathogenesis and molecular and phenotypic AMR determinants, and the development of new drugs and diagnostic technologies [6–8,57–59], but the pipeline remains relatively sparsely filled.

Research regarding improved prevention should also be promoted. One new approach includes the use of antiseptic mouthwash against gonococci in pharyngeal infection [60]. Appropriate uptake of an effective gonococcal vaccine would likely be the only sustainable way to control gonorrhea. There are major challenges in the development of an effective gonococcal vaccine; however, in recent years, substantial progress has been made to address these challenges, and increased vaccine research should be a high priority [61,62].

Furthermore, several novel gonorrhea antimicrobials are in the advanced stages of clinical trial evaluation, including solithromycin (CEM-101), gepotidacin (GSK2140944), and zoliflodacin (AZD0914/ETX0914). Several additional new antimicrobials have shown potent in vitro activity against gonococci, but clinical data are lacking. Several older antimicrobials such as spectinomycin, gentamicin, ertapenem, and fosfomycin have also been suggested for future treatment of gonorrhea [6–8]. Since monotherapy of gonorrhea is no longer a viable option in many settings worldwide, any new antimicrobial should be considered as a part of a dual-antimicrobial regimen.

Use of newer molecular diagnostic technologies can also reduce inappropriate treatment and slow the spread of AMR. Additional research is imperative to identify new AMR determinants (and ideally their induction/selection, evolution and biological fitness) and to evaluate how molecular AMR assays can supplement phenotypic AMR surveillance, for example, by significantly increasing the sample size in surveillance. Ultimately, novel rapid molecular point-of-care gonococcal tests, which include AMR prediction, will guide personalized treatment at the first health-care visit [6,57–59]. These advances will enhance the management and public health control of both gonorrhea and gonococcal AMR. At present, no commercial gonococcal NAATs detecting AMR determinants are available, but many in-house NAATs have been developed. Nevertheless, shortcomings of genetic AMR prediction need to be considered, such as suboptimal sensitivity and specificity in their prediction of AMR, cross-reactions with other bacteria and, critically, the fact that novel AMR determinants will not be detected [6,57–59]. The next-generation diagnostics are now incorporating new technologies to miniaturize devices and detect gonococci at the point of care. In the near future, these point-of-care tests might also be able to incorporate detection of AMR determinants; consequently, antimicrobials that are no longer recommended, such as ciprofloxacin, could be effectively used for at least some patients to spare first-line antimicrobials [57–59]. The cost-effectiveness of these new point-of-care tests and their accessibility in less-resourced settings need to be ensured. Finally, high-throughput genomics is revolutionizing our understanding of AMR and the spread of AMR gonococcal strains, as well as research aimed at improving diagnostics, AMR prediction, and vaccine development [20,63–71].

## Conclusions

Gonococcal AMR is a major concern that threatens our ability to treat and control gonorrhea, and its complications and sequelae, globally. WHO GASP, in collaboration with other GASPs, monitors gonococcal AMR worldwide. However, GASPs need to be substantially strengthened, especially in many less-resourced settings in Eastern Europe, Central Asia, parts of Latin America (including the Caribbean), the WHO Eastern Mediterranean Region and the WHO African Region. Building national leadership and commitment—both political and financial—to address gonococcal AMR is essential. In general, there is a need for greater involvement and increasing country-level ownership through advocacy, capacity-building, strengthened communications, and linkages with broader interventions to control AMR. Efforts are underway to ensure that gonococcal AMR is addressed in the global AMR action plan [16] and in the global AMR research and development agenda. Approaches are also under development to monitor gonococcal AMR within the Global AMR Surveillance System (GLASS; http://www.who.int/antimicrobial-resistance/global-action-plan/surveillance/glass/en/), to conduct regular systematic reviews, and to update treatment guidelines more rapidly. Furthermore, work is ongoing internationally to improve the regulation of drugs and prescription policies and to increase awareness about the correct use of antimicrobials. At the national level, countries need to strengthen and support their gonococcal AMR surveillance programs in the context of national AMR programs. Countries in less-resourced settings should be supported technically and funded appropriately to develop and/or enhance their national GASP and ensure timely dissemination of data as a basis for the revision of treatment guidelines and public health action. Consistent national and international financial and political commitment is urgently needed.

The development of novel antimicrobials for treatment of gonorrhea should be the highest priority. To facilitate the development, clinical evaluation, and registration of new antimicrobials and therapeutic regimens for gonorrhea, WHO has liaised with the Global Antibiotic Research and Development Partnership (GARDP; http://www.dndi.org/diseases-projects/gardp/). In conjunction with the development of novel antimicrobials, strategies to conserve these new antimicrobials while making them accessible should be implemented.

Finally, prevention of gonorrhea remains a paramount public health priority globally. Efforts towards the development of a gonococcal vaccine in conjunction with early detection and screening strategies (including for asymptomatic cervical, anogenital, and pharyngeal infections), partner management (notification and treatment), development of new diagnostics (including point-of-care tests that ideally also detect AMR), and novel treatment options for gonorrhea should be given the greatest attention in order to achieve a 90% reduction of gonorrhea incidence by 2030, relative to the incidence in 2018 [14].

## Supporting information

S1 Table Regional coordinating centers and partners in the WHO Global Gonococcal Antimicrobial Surveillance Programme (WHO GASP). (DOCX)

S2 Table Number of countries reporting data on *Neisseria gonorrhoeae* antimicrobial susceptibility to extended-spectrum cephalosporin, azithromycin (macrolide), or ciprofloxacin (fluoroquinolone) from 2009–2014. The WHO regions are ordered based on estimated gonorrhea burden [1].
(DOCX)

S1 Text Methodologies in gonococcal antimicrobial surveillance programs (GASPs). (DOCX)

## Abbreviations

AMR: antimicrobial resistance
DALY: disability-adjusted life year
EGASP: enhanced GASP
EQA: external quality assurance
ESC: extended-spectrum cephalosporin
GASP: Gonococcal Antimicrobial Surveillance Programme
GLASS: Global AMR Surveillance System
GARDP: Global Antibiotic Research and Development Partnership
LAC: Latin American country
MIC: minimum inhibitory concentration
MSM: men who have sex with men
NAAT: nucleic acid amplification test
QA: quality assurance
QC: quality control
STI: sexually transmitted infection
UN: United Nations
WHO: World Health Organization
YLD: years lived with disability
