# Supplementary material for: Antimicrobial resistance in Neisseria gonorrhoeae: Global surveillance and a call for international collaborative action
Source: PLoS Med. 2017 Jul 7;14(7):e1002344. doi: 10.1371/journal.pmed.1002344 (PMC5501266; doi:10.1371/journal.pmed.1002344)
Supplement: S1 Table — (DOCX) [file pmed.1002344.s001.docx]

**S1 Table. Regional coordinating centres and partners in the WHO Global Gonococcal Antimicrobial Surveillance Programme (WHO GASP).**

| **WHO regions** | **Regional WHO GASP coordinating centers and/or partner reference laboratories** | **No. of countries reporting at least one data point, 2009–2014^a^** |
| --- | --- | --- |
| African Region | - Sexually Transmitted Infections Reference Centre, National Institute of Communicable Diseases, National Health Laboratory Service, Johannesburg, South Africa - Unité des Agents des IST, Département de Bactériologie-Virologie, Institut Pasteur de Côte d’Ivoire, Abidjan, Côte d’Ivoire - Institute of Clinical Research, University of Nairobi, Nairobi, Kenya | 9/47 (19%) |
| Region of the Americas | - Sexually Transmitted Infections Reference Centre, National Institute of Infectious Disease, Buenos Aires, Argentina - University of Saskatchewan, Saskatchewan, Saskatoon, Canada - Public Health Agency of Canada, Winnipeg, Manitoba, Canada - Sexually Transmitted Disease Prevention Program, National Center for HIV/AIDS, Viral Hepatitis, STD and TB Prevention, Centers for Disease Control and Prevention, Atlanta, GA, USA | 16/35 (46%) |
| Eastern Mediterranean Region | - STD Laboratory, Bacterial Department, National Institute of Hygiene, Rabat, Morocco | 3/22 (14%) |
| European Region | - WHO Collaborating Centre for Gonorrhoea and other STIs, National Reference Laboratory for Pathogenic Neisseria, Örebro University Hospital, Örebro, Sweden - Antimicrobial Resistance and Healthcare Associated Infections Unit, Public Health England, Colindale, London, United Kingdom - European Centre for Disease Prevention and Control, Stockholm, Sweden | 27/54 (50%) |
| South-East Asian Region | - WHO GASP South-East Asian Regional Reference Laboratory, VMMC and Safdarjung Hospital, New Delhi, India | 6/11 (54%) |
| Western Pacific Region | - WHO Collaborating Centre for STDs, South Eastern Area Laboratory Services (SEALS), The Prince of Wales Hospital, Sydney, Australia | 17/29 (59%) |

^a^ The number of countries that reported the susceptibility of at least one antimicrobial for at least one year from 2009 to 2014.
