# Supplementary material for: Antimicrobial resistance in Neisseria gonorrhoeae: Global surveillance and a call for international collaborative action
Source: PLoS Med. 2017 Jul 7;14(7):e1002344. doi: 10.1371/journal.pmed.1002344 (PMC5501266; doi:10.1371/journal.pmed.1002344)
Supplement: S2 Table — The WHO regions are ordered based on estimated gonorrhea burden [1]. (DOCX) [file pmed.1002344.s002.docx]

**S2 Table: Number of countries reporting data on *Neisseria gonorrhoeae* antimicrobial susceptibility to extended-spectrum cephalosporin, azithromycin (macrolide) or ciprofloxacin (fluoroquinolone) from 2009–2014. The WHO regions are ordered based on estimated gonorrhoea burden[1].**

| **WHO Region** | **2009** | **2010** | **2011** | **2012** | **2013** | **2014** |
| --- | --- | --- | --- | --- | --- | --- |
| **Western Pacific** | 15 | 17 | 12 | 13 | 13 | 11 |
| **South-East Asia** | 4 | 4 | 6 | 4 | 4 | 6 |
| **Africa** | 3 | 3 | 2 | 1 | 2 | 2 |
| **Americas** | 13 | 10 | 8 | 11 | 9 | 9 |
| **Europe** | 19 | 24 | 23 | 23 | 22 | 23 |
| **Eastern Mediterranean** | 2 | 0 | 1 | 1 | 1 | 1 |
| **Total** | 56 | 58 | 52 | 53 | 51 | 52 |

Reference:

1. Newman L, Rowley J, Vander Hoorn S, Wijesooriya NS, Unemo M, Low N, et al. Global estimates of the prevalence and incidence of four curable sexually transmitted infections in 2012 based on systematic review and global reporting. PloS One. 2015; 10(12):e0143304. doi: 10.1371/journal.pone.0143304
