# Supplementary material for: Antimicrobial resistance in Neisseria gonorrhoeae: Global surveillance and a call for international collaborative action
Source: PLoS Med. 2017 Jul 7;14(7):e1002344. doi: 10.1371/journal.pmed.1002344 (PMC5501266; doi:10.1371/journal.pmed.1002344)
Supplement: S1 Text — (DOCX) [file pmed.1002344.s003.docx]

**S1 Text: Methodologies in gonococcal antimicrobial surveillance programmes (GASPs)**

The 2012 WHO Surveillance Standards recommend the use of appropriate sampling methods, laboratory techniques and quality assurance (QA) procedures [1-3]. These standards include the microbiological and epidemiologic requirements to ensure data validity and comparability. Due to the highly variable nature of *Neisseria gonorrhoeae*, these standards and other similar standards need continuous and regular reviewing and, when required, updating.

Different countries vary significantly in their approaches to gonococcal AMR surveillance. Some regions or countries conduct AMR surveillance by testing all *N. gonorrhoeae* isolates (e.g., Australia [4]); through sentinel sites with a pre-defined, representative sample size on a monthly basis (e.g., US GISP [8-9]); or with a pre-defined, representative sample size using isolates collected continuously for several months until the sample size has been reached (e.g., Euro-GASP [10-11,8] and UK GRASP [12]). However, the majority of countries conduct passive and voluntary AMR surveillance based on submitted laboratory data or *ad hoc* surveys every 2–3 years. Very few GASPs, e.g., Euro-GASP and US GISP, collect comprehensive epidemiologic data for the patients and link it to the AMR data [8-11].

In the GASPs, the laboratory testing is either centralized through reference laboratories or decentralized, and conducted at the site of collection. There are a number of laboratory methods used for AMR testing, and they vary in their interpretative criteria. In countries participating in Euro-GASP [10-11],US GISP [8-9], Canada [13] and Australia [4], only quantitative methods for determination of the minimum inhibitory concentration (MIC) of antimicrobials, such as agar dilution method or Etest, are used. However, in some countries or regions, for some antimicrobials, only the agar dilution breakpoint method is used. Qualitative disc diffusion methods, with dichotomous interpretative criteria, have been widely used in the GASPs in the WHO South-East Asian Region [14], the Western Pacific Region [15], and in the few Eastern Mediterranean Region countries that have performed any gonococcal AMR surveillance. It is recommended that, at a minimum, decreased susceptibility or resistance to ESCs identified with disc diffusion methods should be verified with quantitative MIC determination, and the use of quantitative MIC determination methods is being expanded through GASP initiatives.

Most countries use either the interpretative criteria of the European Committee on Antimicrobial Susceptibility Testing (EUCAST; www.eucast.org) or the Clinical Laboratory and Standards Institute (CLSI; www.clsi.org).

To harmonize, standardize and quality assure the laboratory methodologies, and for comparability of GASP data globally, the WHO GASP develop panels of WHO *N. gonorrhoeae* reference strains for quality control (QC) and QA of the AMR data nationally and internationally. WHO *N. gonorrhoeae* reference strains (n=8) were selected and characterized in detail – phenotypically and genetically – in 2008 by the regional coordinating laboratories for the WHO Western Pacific Region and the European Region [1]. In 2016, a new panel of WHO *N. gonorrhoeae* reference strains, with six additional strains and further phenotypic and molecular (including whole-genome sequencing) characterization, was developed [2]. These WHO *N. gonorrhoeae* reference strains enable intra-laboratory and inter-laboratory comparison of test results at adjoining national or regional centres, with variability in laboratory methods [1,2]. These WHO reference strains can also be used for QC in phenotypic and molecular diagnostics, for molecular AMR prediction and molecular epidemiology, and as fully characterized reference genomes in, for example, whole-genome sequencing analysis [2].

**References:**

1. Unemo M, Fasth O, Fredlund H, Limnios A, Tapsall J. Phenotypic and genetic characterization of the 2008 WHO *Neisseria gonorrhoeae* reference strain panel intended for global quality assurance and quality control of gonococcal antimicrobial resistance surveillance for public health purposes. J Antimicrob Chemother. 2009; 63(6):1142-1151. doi: 10.1093/jac/dkp098
2. Unemo M, Golparian D, Sánchez-Busó L, Grad Y, Jacobsson S, Ohnishi M, et al. The novel 2016 WHO *Neisseria gonorrhoeae* reference strains for global quality assurance of laboratory investigations: Phenotypic, genetic and reference genome characterization. J Antimicrob Chemother. 2016; 71(11):3096-3108. doi: 10.1093/jac/dkw288
3. UNAIDS/WHO Working Group on Global HIV/AIDS and STI Surveillance. Strategies and laboratory methods for strengthening surveillance of sexually transmitted infections 2012. Geneva: World Health Organization; 2012. Available from: <http://apps.who.int/iris/bitstream/10665/75729/1/9789241504478_eng.pdf>
4. Lahra MM; Australian Gonococcal Surveillance Programme. Australian Gonococcal Surveillance Programme annual report, 2014. Commun Dis Intell Q Rep. 2015; 39(3):E347-354.
5. Dillon JA, Trecker MA, Thakur SD; Gonococcal Antimicrobial Surveillance Program Network in Latin America and the Caribbean 1990–2011. Two decades of the gonococcal antimicrobial surveillance program in South America and the Caribbean: Challenges and opportunities. Sex Transm Infect. 2013; 89(Suppl 4):iv36-iv41. doi: 10.1136/sextrans-2012-050905
6. Thakur SD, Araya P, Borthagaray G, Galarza P, Hernandez AL, Payares D, et al. Resistance to Ceftriaxone and Azithromycin in Neisseria gonorrhoeae Isolates From 7 Countries of South America and the Caribbean: 2010–2011. Sex Transm Dis. 2017; 44(3):157-160. doi: 10.1097/OLQ.0000000000000587
7. Ndowa FJ, Francis JM, Machiha A, Faye-Kette H, Fonkoua MC. Gonococcal antimicrobial resistance: Perspectives from the African region. Sex Transm Infect. 2013; 89(Suppl 4):iv11-iv15. doi: 10.1136/sextrans-2012-050907
8. Kirkcaldy RD, Kidd S, Weinstock HS, Papp JR, Bolan GA. Trends in antimicrobial resistance in Neisseria gonorrhoeae in the USA: The Gonococcal Isolate Surveillance Project (GISP), January 2006 – June 2012. Sex Transm Infect. 2013; 89(Suppl 4):iv5-10. doi: 10.1136/sextrans-2013-051162
9. Kirkcaldy RD, Harvey A, Papp JR, Del Rio C, Soge OO, Holmes KK, et al. Neisseria gonorrhoeae antimicrobial susceptibility surveillance – The Gonococcal Isolate Surveillance Project, 27 sites, United States, 2014. MMWR Surveill Summ. 2016; 65 (7):1-19. doi: 10.15585/mmwr.ss6507a1
10. Spiteri G, Cole M, Unemo M, Hoffmann S, Ison C, van de Laar M. The European Gonococcal Antimicrobial Surveillance Programme (Euro-GASP) – a sentinel approach in the European Union (EU)/European Economic Area (EEA). Sex Transm Infect. 2013; 89(Suppl 4):iv16-iv18. doi: 10.1136/sextrans-2013-051117
11. Cole MJ, Spiteri G, Jacobsson S, Pitt R, Grigorjev V, Unemo M; Euro-GASP Network. Is the tide turning again for cephalosporin resistance in Neisseria gonorrhoeae in Europe? Results from the 2013 European surveillance. BMC Infect Dis. 2015; 15(1):321. doi: 10.1186/s12879-015-1013-x
12. Town K, Obi C, Quaye N, Chisholm S, Hughes G; GRASP Collaborative Group. Drifting towards ceftriaxone treatment failure in gonorrhoea: Risk factor analysis of data from the Gonococcal Resistance to Antimicrobials Surveillance Programme in England and Wales. Sex Transm Infect. 2017; 93(1):39-45. doi: 10.1136/sextrans-2016-052583
13. Martin I, Sawatzky P, Liu G, Allen V, Lefebvre B, Hoang L, et al. Decline in decreased cephalosporin susceptibility and increase in azithromycin resistance in Neisseria gonorrhoeae, Canada. Emerg Infect Dis. 2016; 22(1):65-67. doi: 10.3201/eid2201.151247
14. Bala M, Kakran M, Singh V, Sood S, Ramesh V; Members of WHO GASP SEAR Network. Monitoring antimicrobial resistance in Neisseria gonorrhoeae in selected countries of the WHO South-East Asia Region between 2009 and 2012: A retrospective analysis. Sex Transm Infect. 2013; 89(Suppl 4):iv28-35. doi: 10.1136/sextrans-2012-050904
15. Lahra MM, Lo YR, Whiley DM. Gonococcal antimicrobial resistance in the Western Pacific Region. Sex Transm Infect. 2013; 89(Suppl 4):iv19-23. doi: 10.1136/sextrans-2012-050906
